# Monitoring Changes in the Antimicrobial-Resistance Gene Set (ARG) of Raw Milk and Dairy Products in a Cattle Farm, from Production to Consumption

**DOI:** 10.3390/vetsci11060265

**Published:** 2024-06-08

**Authors:** Ádám Kerek, Virág Németh, Ábel Szabó, Márton Papp, Krisztián Bányai, Gábor Kardos, Eszter Kaszab, Krisztina Bali, Zoltán Nagy, Miklós Süth, Ákos Jerzsele

**Affiliations:** 1 Department of Pharmacology and Toxicology, University of Veterinary Medicine, István utca 2, H-1078 Budapest, Hungary; nemeth.virag@student.univet.hu (V.N.); szabo.abel@student.univet.hu (Á.S.); banyai.krisztian@univet.hu (K.B.); jerzsele.akos@univet.hu (Á.J.); 2 National Laboratory of Infectious Animal Diseases, Antimicrobial Resistance, Veterinary Public Health and Food Chain Safety, University of Veterinary Medicine Budapest, István utca 2, H-1078 Budapest, Hungary; papp.marton@univet.hu (M.P.); kg@med.unideb.hu (G.K.); kaszab.eszter@univet.hu (E.K.); bali.krisztina@univet.hu (K.B.); suth.miklos@univet.hu (M.S.); 3 Centre for Bioinformatics, University of Veterinary Medicine, István utca 2, H-1078 Budapest, Hungary; 4 Veterinary Medical Research Institute, HUN-REN, Hungária krt. 21, H-1143, Budapest, Hungary; 5 One Health Institute, University of Debrecen, Nagyerdei krt. 98, H-4032 Debrecen, Hungary; 6 National Public Health Center, Albert Flórián út 2-6, H-1097 Budapest, Hungary; 7 Department of Gerontology, Faculty of Health Sciences, University of Debrecen, Sóstói út 2-4, H-4400 Nyiregyhaza, Hungary; 8 Department of Microbiology and Infectious Diseases, University of Veterinary Medicine, István utca 2, H-1078 Budapest, Hungary; 9 Biological Research and Development Department, CEVA-Phlyaxia Zrt., Szállás utca 5, H-1107 Budapest, Hungary; zoltan.nagy@ceva.com; 10 Institute of Food Chain Science, University of Veterinary Medicine, István utca 2, H-1078 Budapest, Hungary

**Keywords:** antimicrobial-resistance genes, raw milk, dairy products, next-generation sequencing, NGS, cattle, food safety, public health, ESBL, taxonomy

## Abstract

**Simple Summary:**

In the 21st century, one of society’s most essential needs is safe food. This need is closely tied to understanding risks that affect both animals and humans. A major global concern is the spread of antibiotic resistance through food, such as raw milk and dairy products. In our research, we aim to track changes in antibiotic-resistant genes in raw milk and products made from raw milk. Our study follows the journey of milk “from farm to fork”, starting at a small dairy farm, through processing, and finally to the consumer, using advanced genetic testing. We identified 112 antibiotic-resistance genes in total. We found that raw cheese had fewer resistant genes compared to raw milk. However, after one month of aging, the number of resistant genes in the cheese increased significantly, even surpassing the initial levels found in raw milk. This is particularly concerning for public health because of the presence of a highly dangerous type of antibiotic-resistance gene known for causing serious health issues. In conclusion, more research like ours is necessary to understand how antibiotic-resistance genes change during food production. It is also important to assess the risk of these genes spreading through raw food products and to ensure that food safety standards are maintained. A new approach to food safety, using advanced genetic testing, should be considered.

**Abstract:**

Raw milk and dairy products can serve as potential vectors for transmissible bacterial, viral and protozoal diseases, alongside harboring antimicrobial-resistance genes. This study monitors the changes in the antimicrobial-resistance gene pool in raw milk and cheese, from farm to consumer, utilizing next-generation sequencing. Five parallel sampling runs were conducted to assess the resistance gene pool, as well as phage or plasmid carriage and potential mobility. In terms of taxonomic composition, in raw milk the Firmicutes phylum made up 41%, while the Proteobacteria phylum accounted for 58%. In fresh cheese, this ratio shifted to 93% Firmicutes and 7% Proteobacteria. In matured cheese, the composition was 79% Firmicutes and 21% Proteobacteria. In total, 112 antimicrobial-resistance genes were identified. While a notable reduction in the resistance gene pool was observed in the freshly made raw cheese compared to the raw milk samples, a significant growth in the resistance gene pool occurred after one month of maturation, surpassing the initial gene frequency. Notably, the presence of extended-spectrum beta-lactamase (ESBL) genes, such as *OXA-662* (100% coverage, 99.3% identity) and *OXA-309* (97.1% coverage, 96.2% identity), raised concerns; these genes have a major public health relevance. In total, nineteen such genes belonging to nine gene families (*ACT*, *CMY*, *EC*, *ORN*, *OXA*, *OXY*, *PLA*, *RAHN*, *TER*) have been identified. The largest number of resistance genes were identified against fluoroquinolone drugs, which determined efflux pumps predominantly. Our findings underscore the importance of monitoring gene pool variations throughout the product pathway and the potential for horizontal gene transfer in raw products. We advocate the adoption of a new approach to food safety investigations, incorporating next-generation sequencing techniques.

## 1. Introduction

The discovery and widespread use of antimicrobial agents has revolutionized modern medicine, fundamentally altered therapeutic protocols and extended the average life expectancy by approximately 23 years [1]. However, the excessive and unregulated use of antibiotics in recent decades, coupled with social and economic trends, has significantly hastened the genetic selection and dissemination of antimicrobial-resistant bacteria. Consequently, human deaths related to antimicrobial resistance (AMR) have seen a dramatic increase, with approximately 700,000 deaths per year currently attributed to AMR [2]. Conservative estimates suggest this figure could soar to as high as 10 million per year by 2050 if antibiotic usage remains unchecked and new therapies and active substances are not rapidly developed [3]. Although the problem’s importance is widely acknowledged, the spread of infections caused by multi-resistant bacteria continues to rise [4,5]. The World Health Organization (WHO) is actively working to curb the spread of AMR through stringent regulations, alongside promoting targeted and judicious antibiotic use. Achieving this goal necessitates coordinated efforts across human and veterinary medicine, as well as environmental management. In veterinary medicine and agriculture, the reduction or elimination of antibiotics crucial for human medicine (e.g., 3rd and 4th generation cephalosporins, fluoroquinolones, colistin) has become imperative, and the use of antibiotics for growth promotion has been banned for decades [6].

In the cattle-farming sector, the most common indication for antibiotic use is the treatment of inflammatory diseases, with mastitis [7] and the bovine respiratory disease syndrome (BRD) in beef cattle all over the world being the primary cause [8]. Masebo et al. showed that BRD is the most common animal welfare issue leading to antibiotic use among beef cattle [9]. Recent studies have highlighted a concerning increase in antimicrobial resistance within the cattle sector, with percentages rising from 7% to 63% for penicillin, 0% to 12% for oxacillin, 0% to 93% for erythromycin, 0% to 28% for tetracycline and 4.5% to 7.5% for sulfadimethoxin in recent decades [10,11,12,13,14,15,16]. The microbiota of products of animal origin can come into direct contact with human bacteria, and ARGs accumulated from high levels of antibiotics can be transferred to the human microbiota via animal products [17].

The demand for unpasteurized, or raw, milk and dairy products has surged over the last two decades due to a perception of improved flavor and nutritional benefits. Surveys indicate that approximately 3% of the US population consumes unpasteurized milk, with a growing demand for its legal sale. However, consuming raw dairy products poses serious health risks as it can transmit both milk-borne diseases and antimicrobial-resistance genes (ARGs). Raw milk exhibits significantly higher ARG counts than pasteurized milk [18]. A strong correlation has been shown between the consumption of raw milk containing various pathogens (including human *Campylobacter* spp., *Salmonella* Typhimurium, Shiga toxin-producing *Escherichia coli* (STEC), tick-borne encephalitis virus (TBEV), *Brucella melitensis* and *Mycobacterium bovis*) and severe health consequences for affected individuals [19]. According to the European Union One Health 2020 Zoonoses Report, there were 325 reported cases of illness linked to milk, cheese and other dairy products, with 67 cases requiring hospitalization and one fatality in the EU that year [20].

Investigations into the ARG gene pool in raw milk have revealed a correlation between antibiotic use and the prevalence of resistant Gram-negative bacteria. The presence of *Pseudomonas* spp. emerges as a significant factor in ARG carriage in raw milk, alongside lactic acid bacteria such as *Lactobacillus* spp. and *Lactococcus* spp. [21].

The aim of this study was to track the changes in the microbiome composition of raw milk and cheese from production to consumption, with a specific focus on their antimicrobial-resistance gene pool, plasmid or phage carriage, and mobility. The hypothesis tested was whether these products pose a risk for the potential spread of antimicrobial resistance.

## 2. Materials and Methods

### 2.1. Samples Origin and Collection

The samples originated from a small dairy farm with 30 cows located in Körösladány, Hungary. During the sampling process, the entire trajectory of the starting batch was meticulously followed. To ensure statistical comparability, five replicate samples were taken at each sampling point. The initial sampling was conducted on the bulk raw milk (30 mL/sample), from which the cheese is produced. This milk undergoes curdling using a proprietary curdling agent and a specialized rennet product (CHY MAX PLUS 17 mL/100 mL). Subsequently, five samples of fresh cheese (30 g/sample) were obtained post-curdling, representing the next critical point for analysis.

Following this, five additional samples were collected after the cheese had undergone one month of maturation (30 g/sample). The maturation process took place under controlled conditions of 17 °C and 70% humidity, utilizing pine-wood shelves. For storage, the samples were placed in 50 mL centrifuge tubes (VWR International Ltd., Debrecen, Hungary), each individually labeled, and stored in an ultra-low refrigerator at −80 °C until processing. Personal transport of the samples was carried out promptly after each sampling event.

### 2.2. DNA Extraction, Sequencing

To extract nucleic acid from the bacteria, we utilized the QIAamp DNeasy PowerFood Microbial kit (100), cat.no. 21000-100 kit (Qiagen, Hilden, Germany), following the manufacturer’s protocol. For milk samples, 1.8 mL was used as per the protocol, while for cheeses, 0.4 g was crushed with a sterile scalpel and supplemented with 1 mL of sterile phosphate buffer solution. Subsequently, the samples were shaken in a Qiagen TissueLyser LT (Qiagen GmbH, QIAGEN Strasse 1, 40724 Hilden, Germany) at 50 Hz for 10 min, followed by elution of each sample at 50 µg/mL concentration. Fluorometric quantification was performed using the Qubit^®^ dsDNA BR Assay kit (Thermo Fisher SSC, Budapest, Hungary).

Paired-end reads from DNA were analyzed using the Illumina NextSeq 500 sequencer (Illumina, San Diego, CA, USA) [22]. DNA libraries were constructed using the Illumina^®^ Nextera XT DNA Library Preparation Kit (Illumina, San Diego, CA, USA), with indexes used to label DNA fragments from the Nextera XT Index Kit v2 Set C (Illumina, San Diego, CA, USA).

In the initial step, amplified cDNA samples were diluted to a concentration of 0.2 ng/µL in a final volume of 2.5 µL. Subsequently, they were mixed with 5 µL of Tagment DNA buffer (Illumina, San Diego, CA, USA) and 2.5 µL of AmpliconTagment Mix reagent (Illumina, San Diego, CA, USA) for the tagmentation reaction. The reaction mixture was then incubated at 55 °C for 6 min in a GeneAmp PCR System 9700 (Applied Biosystems, Waltham, MA, USA) and cooled to 10 °C. Neutralize Tagment buffer (Illumina, San Diego, CA, USA) (2.5 µL) was added and incubated for 5 min at room temperature.

To prepare the DNA library, 7.5 µL of Nextera PCR Master mix (Illumina, San Diego, CA, USA) was mixed with 2.5–2.5 µL of i5 and i7 index primers, added to the tagged DNA sample and amplified by PCR. The PCR comprised 12 cycles (95 °C for 10 s, 55 °C for 30 s, 72 °C for 30 s) following an initial denaturation at 95 °C for 30 s. This was followed by a final elongation step at 72 °C for 5 min, and then the samples were cooled to 10 °C.

The resulting indexed DNA library was purified using the Gel/PCR DNA Fragments Extraction kit (Geneaid Biotech, Xinpei, Taiwan) following the column purification protocol. Finally, the Qubit^®^ dsDNA HS Assay kit (Thermo Fisher Scientific, Waltham, MA, USA) was employed for fluorometric quantification to ensure the samples were of the same standard.

### 2.3. Bioinformatics Analysis

Quality control of the raw sequences was conducted using FastQC v0.11.9 [23] software, with unsatisfactory quality sections filtered out using Trim-Galore v0.6.7 [24]. The host genome, specifically the Bos taurus genome (ARS-UCD1.3) sequences, was filtered out using Bowtie2 [25] with high sensitivity settings to minimize false positive rates [26].

Subsequently, the read sequences were assembled into longer sequences (contigs) using MEGAHIT v1.2.9 [27] software. From these contigs, all possible Open Reading Frames (ORFs) were determined using Prodigal v2.6.3 [28]. Protein sequences were derived in base order and compared with sequences in The Comprehensive Antibiotic Resistance Database (CARD) [29] using Resistance Gene Identifier (RGI) v6.0.0 software (downloaded 01 September 2023). Only results meeting the strict rating threshold defined in the CARD database were retained.

We examined the mobility of resistance genes using MobileElementFinder v1.1.2 software [30], which predicts Mobile Genetic Element (MGE) genes on contigs. Resistance genes were considered potentially mobile if they were located within the median distance of the longest composite transposon in the MGE database on the contig. Carriage on plasmids was assessed using PlasFlow v1.1 software, while the presence of phage genomes on contigs was investigated using VirSorter v2.2.3 [31] software.

Each identified resistance gene’s significance was compared with information in the CARD database, considering data with over 60% coverage and sequence identity. From this database, we determined the resistance mechanism caused by each resistance gene and to which groups of antibiotics the presence of the gene confers resistance [29].

Principal component analysis was employed to assess the expressiveness of the received ARG samples and their coverage of the entire ARG repertoire [32]. Genes with at least 90% coverage and identity were depicted on a heatmap. Logistic regression analysis was used to examine the likelihood of differences in ARG mobility among various groups. Logistic regression analysis is a statistical method used to model the relationship between a binary dependent variable and one or more independent variables, estimating the probability of a binary outcome [33]. Shannon diversity at the genus and species levels between individual samples was investigated using the Mann-Whitney test. Mann-Whitney test is a non-parametric test used to compare differences between two independent groups when the dependent variable is either ordinal or continuous, but not normally distributed [34,35]. This test measures biodiversity, where a higher Shannon index indicates greater diversity of genera or species within the samples. Furthermore, the distribution of genera and species was examined using the same test. This provides information on the abundance of instances from specific genera or species within a given sample, allowing inference about the ecological or biochemical characteristics of the samples. Differences between samples were analyzed using Bray-Curtis distances and non-metric multidimensional scaling (NMDS) ordination, with the PERMANOVA test (permutational multivariate analysis of variance) applied to test the significance of group differences in the microbial communities. Bray-Curtis dissimilarity is a measure used to quantify the compositional dissimilarity between two different sites or samples, based on counts of species or other units. NMDS is an ordination technique that reduces the dimensionality of multivariate data to visualize differences and patterns between samples. PERMANOVA is a statistical test used to compare the differences between groups of multivariate samples based on permutation methods [36]. The results were statistically analysed using the R program version 4.3.0 [37].

## 3. Results

### 3.1. The Gene Pool Identified from the Samples

A total of 112 ARGs were identified in the samples, and their details are provided in the Supplementary Tables S1–S5, including their identity, degree of coverage, associated antibiotic group and resistance mechanism.

One of the most crucial resistance mechanisms from a public health standpoint is the production of β-lactamase, responsible for enzymatic inactivation. Nineteen genes, belonging to nine gene families (*ACT*, *CMY*, *EC*, *ORN*, *OXA*, *OXY*, *PLA*, *RAHN*, *TER*) associated with various levels of β-lactamase production were identified. Notably, one gene family (*OXA*) was responsible for widespread β-lactamase production, with *OXA-662* detected in the raw milk sample and *OXA-309* in the fresh cheese sample.

The fluoroquinolone-specific efflux pump gene *abaQ* was exclusively detected in raw milk samples, while *emrA*, *emrB*, *emrR*, *mdtH*, *patA* and *patB* were present in almost all samples throughout the product pathway. The *marA* gene, responsible for reduced permeability and efflux pump determination, was present in all samples, whereas *ramA* was solely detected in raw milk and mature cheese samples. Additionally, multidrug-resistance genes *acrAB-tolC* and *soxR*, responsible for the efflux pump system and target modification, were present in all samples, alongside the permeability-reducing gene *soxS*.

Among the *AAC*(*6*′) gene family responsible for aminoglycoside inactivation, *AAC*(*6*′)*-If* was detected in raw milk and mature cheese samples, while *AAC*(*6*′)*Ii* and *AAC*(*6*′)*-Iih* were only observed in mature cheese, possibly indicating product contamination. Other notable findings include the presence of *aadA27*, *APH*(*3*″)*-Ib*, *APH*(*6*)*-Id* and *ANT(3″)-IIc* genes mainly in raw milk.

A significant number of genes responsible for target modification against peptide antibiotics were detected in all samples, with some, like *arnT*, *bacA*, *eptB*, *ICR-Mo* and *pmrF*, present in both raw milk and mature cheese, while others like *eptA* and *ugd* were exclusively found in mature cheese. The *ompA* gene, determining reduced permeability, was identified solely in raw milk samples, whereas the efflux pump gene *yojI* was exclusively detected in mature cheese. Among the identified genes, 57 were plasmid-borne and seven were phage-borne. Additionally, 15 genes associated with mobile genetic elements (MGEs) were found, with some appearing on plasmids in one sample and as phage-borne in another. Notably, genes such as *APH*(*3*″)*-Ib* and *APH(6)-Id*, responsible for aminoglycoside resistance, were detected only in raw milk samples. However, the sul1 and *tetM* genes, which confer resistance to sulfonamide and tetracycline drugs, respectively, were exclusively found in mature cheese samples. The MGE *qacEdelta1* gene, conferring resistance to disinfectants, was also observed on a plasmid in mature cheese samples.

During principal component analysis, the majority of samples were within the low to medium value range (Supplementary Figure S1), indicating a minor contribution to explaining the variances between samples or differences along the given dimension. The heatmap representation of ARGs (Supplementary Figure S2) showed Z-scores between −2 and +2, suggesting diverse and varied ARG values across the three main product types. Similar analyses were conducted solely on significant (*p* < 0.05) genes, i.e., those determining antimicrobial-resistance patterns. However, significant differences were not observed during principal component analysis (Supplementary Figure S3) or in the heatmap (Supplementary Figure S4). During logistic regression analysis, the effects of samples were adjusted considering them as random factors. Supplementary Table S6 illustrates the regression results, where the intercept represents the raw milk category, and the estimated parameters denote the Odds Ratios (ORs) for gene mobility within the respective group (fresh or matured cheese) compared to the reference category (raw milk). An OR greater than 1 indicates higher probability of mobility, while an OR less than 1 indicates a lower chance in the respective category. At the genus (Supplementary Figure S5) and species (Supplementary Figure S8) levels, the distribution of Shannon diversity between samples revealed significant differences among all groups. Raw milk exhibited high values, while lower values were observed in fresh and matured cheese produced from the raw milk, indicating a narrowing of genus biodiversity. The distribution of genera (Supplementary Figure S6) and species (Supplementary Figure S9) was highest in raw milk, moderate in fresh cheese and lowest in matured cheese. At the genus (Supplementary Figure S7) and species (Supplementary Figure S10) levels, the results of Bray-Curtis distances between samples showed significant differences (*p* = 0.001) with PERMANOVA test assistance.

### 3.2. Frequency of the Identified Genes per Sample

**Figure 1:**
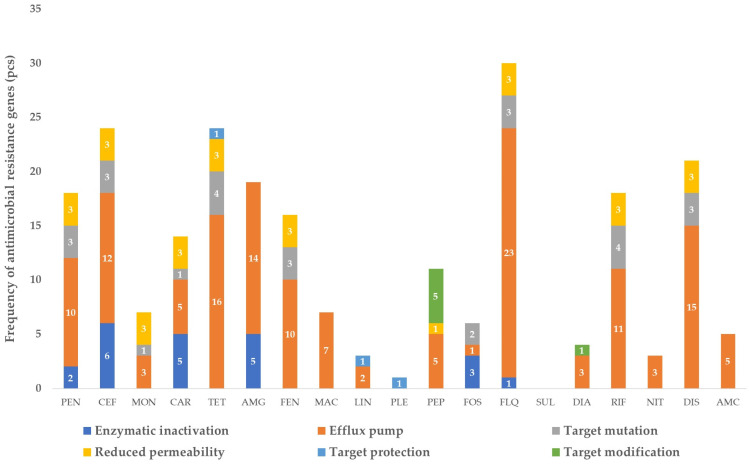
Frequency of antimicrobial-resistance genes identified in raw milk samples, by drug group and resistance mechanism. PEN—penicillins; CEF—cephalosporins; MON—monobactams; CAR—carbapenems; TET—tetracyclines; AMG—aminoglycosides; FEN—phenicols; MAC—macrolides; LIN—lincosamides; PLE—pleuromutilins; PEP—peptide antibiotics; FOS—phosphomicin; FLQ—fluoroquinolones; SUL—sulphonamides; DIA—diaminopyrimidines; RIF—rifamicins; NIT—nitroimidazoles; DIS—disinfectants; AMC—aminocoumarins. The numbers in the bar chart represent the exact number of pieces of each resistance mechanism.

The frequencies of identified genes in raw milk samples, categorized by resistance mechanism and drug class, are summarized in Figure 1. The largest proportion of resistance genes (30) was identified against fluoroquinolone drugs, predominantly comprising efflux pump determinants (23). Of notable concern was the prevalent presence of the extended-spectrum beta-lactamase (ESBL) gene *OXA-662*, which is capable of conferring resistance to penicillins, cephalosporins and carbapenems through enzymatic inactivation. This gene showed full coverage and 99.3% identity.

Regarding peptide drugs critical for public health, a total of five efflux pump determinants (*kpnE*, *kpnF*, *kpnG*, *kpnH*, *lptD*), five target-modification genes (*pmrF*, *arnT*, *bacA*, *eptB*, *ICR-Mo*) and one reduced permeability determinant gene (*ompA*) were observed. Additionally, twenty-one genes responsible for resistance to disinfectants were identified.

Figure 1 provides a comprehensive overview of the distribution of resistance genes in raw milk samples, highlighting the diversity of mechanisms and drug classes involved.

**Figure 2:**
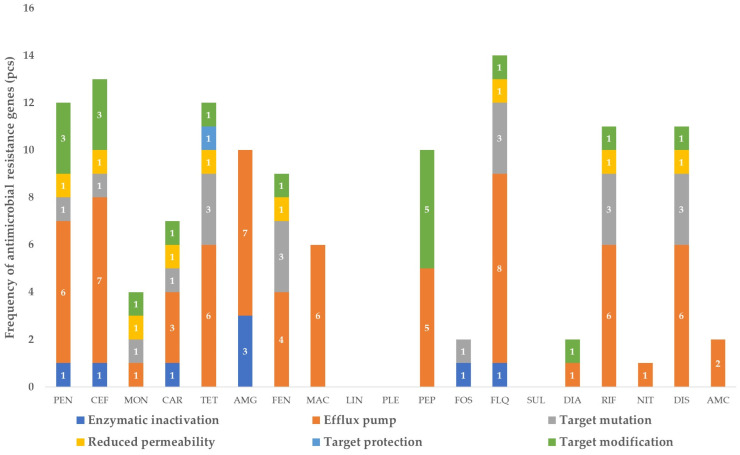
Frequency of antimicrobial-resistance genes identified in fresh cheese samples, by drug group and resistance mechanism. PEN—penicillins; CEF—cephalosporins; MON—monobactams; CAR—carbapenems; TET—tetracyclines; AMG—aminoglycosides; FEN—phenicols; MAC—macrolides; LIN—lincosamides; PLE—pleuromutilins; PEP—peptide antibiotics; FOS—phosphomicin; FLQ—fluoroquinolones; SUL—sulphonamides; DIA—diaminopyrimidines; RIF—rifamicins; NIT—nitroimidazoles; DIS—disinfectants; AMC—aminocoumarins. The numbers in the bar chart represent the exact number of pieces of each resistance mechanism.

In the fresh cheese samples, a notable reduction in the resistome was observed (Figure 2). Despite this reduction, fluoroquinolone-resistance genes remained dominant (14), with efflux pumps comprising the majority (8). Among the ESBL genes, *OXA-309* was identified. Similar to the results from the raw milk samples, genes conferring resistance to peptide drugs via efflux pumps were observed, along with the five genes responsible for target modification. Interestingly, genes conferring resistance to previously observed lincosamides (*lmrD*, *lsaA*, *isaD*, *mdtM*) and pleuromutilins (*isaD*) were not identified in the fresh cheese samples. This suggests a species-level selection of the microbial population during cheese production, leading to a narrowed gene pool.

Figure 2 illustrates the significant changes in the resistome composition between raw milk and fresh cheese, highlighting the impact of the cheese production process on the microbial community and its resistance gene profile.

**Figure 3:**
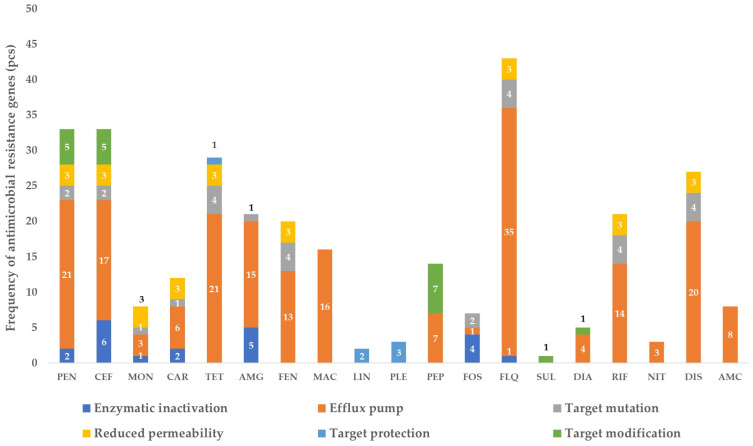
Frequency of antimicrobial-resistance genes identified in matured cheese samples, by drug group and resistance mechanism. PEN—penicillins; CEF—cephalosporins; MON—monobactams; CAR—carbapenems; TET—tetracyclines; AMG—aminoglycosides; FEN—phenicols; MAC—macrolides; LIN—lincosamides; PLE—pleuromutilins; PEP—peptide antibiotics; FOS—phosphomicin; FLQ—fluoroquinolones; SUL—sulphonamides; DIA—diaminopyrimidines; RIF—rifamicins; NIT—nitroimidazoles; DIS—disinfectants; AMC—aminocoumarins. The numbers in the bar chart represent the exact number of pieces of each resistance mechanism.

After the cheese had been matured for one month, a significant enrichment of the resistome was evident (Figure 3). A total of 35 resistance genes associated with fluoroquinolones, predominantly determining efflux pump function, were identified. Additionally, seven genes responsible for efflux pump resistance to peptide antibiotics (*kpnE*, *kpnF*, *kpnG*, *kpnH*, *lptD*, *tolC*, *yojI*) were detected, while the number of genes involved in target modification increased to seven (*arnT*, *bacA*, *eptA*, *aptB*, *ICR-Mo*, *pmrF*, *ugd*).

Notably, genes *AAC*(*6*′)*-Ii* and *AAC*(*6*′)*-Iih*, responsible for enzymatic inactivation of aminoglycosides, were exclusively present in mature cheese samples, indicating likely contamination. Similarly, the presence of genes like *ACT-5*, *EC-14* and *OXY*-type genes, responsible for enzymatic inactivation of penicillins, cephalosporins, carbapenems and other antibiotics, suggests contamination during the maturation process.

Figure 3 illustrates the considerable changes in the resistome composition between fresh cheese and matured cheese samples, underscoring the dynamic nature of resistance gene acquisition and prevalence during cheese aging.

**Figure 4:**
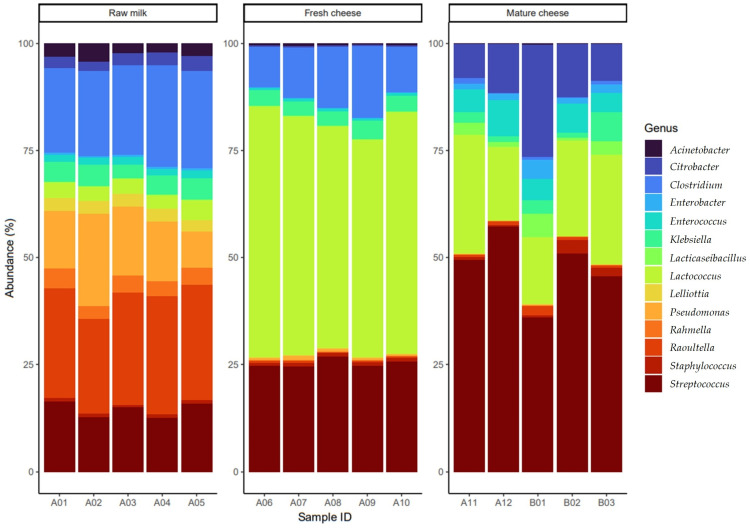
The abundances of core-bacteriome (genera present in at least 10% of samples with a minimum abundance of 1%) varied among samples, including raw milk, raw cheese made from it and cheese examined after one month of aging. It is noticeable that following the inoculation of raw cheese, there is a selection for Gram-positive genera, followed by dominance of the *Streptococcus* genus during maturation. Az űrlap teteje.

In raw milk, it was observed that the *Raoultella* genus (average 24%), *Clostridium* genus (average 20%), *Pseudomonas* genus (average 14%) and *Streptococcus* genus (average 13%) occurred in similar proportions. In contrast, in cheese freshly produced from the raw milk, *Lactococcus* (average 51%) was the dominant genus, with a higher proportion of *Streptococcus* genus (average 24%), while the *Clostridium* genus (average 12%) was slightly lower than in the raw milk. Moreover, in the fresh cheese samples, there was significantly less *Pseudomonas* genus (average 0.7%) and *Raoultella* genus (average 0.4%). However, after a month of aging, *Streptococcus* genus became the dominant bacteria and the proportion of *Citrobacter* genus (average 13%) also increased significantly. At the same time, there was a significant reduction in the proportion of *Lactococcus* genus and a smaller but significant drop in the proportion of *Clostridium* genus (0.5%) in the mature cheese (Figure 4).

## 4. Discussion

The economic significance and importance to public health of the dairy industry is demonstrated by the fact that in the EU in 2021, 10.4 million tons of cheese were produced using 16.4 million tons of skimmed milk and 61.4 million tons of whole milk [38]. The public health risk of consuming raw dairy products is highlighted by the 263 confirmed zoonotic cases identified in Canada between 2005 and 2013 linked to raw dairy consumption, a likely underestimation since most affected individuals do not seek medical attention. Literature suggests the actual number of cases could be 25 times higher [39]. A 2017 study found that unpasteurized milk products were responsible for 840 times more illnesses and 45 times more hospitalizations than pasteurized products [40]. Furthermore, a 2018 study found that raw milk was responsible for nearly three times more hospitalizations than any other foodborne illness [41]. Liu et al. demonstrated the transfer of cephalosporin-resistance genes to *E. coli* strains, which are part of the gut microbiome, from raw milk after ingestion [18]. In the case of raw milk, we detected 31 types of ARGs, while Tóth et al. identified 48 types of ARGs from raw milk [17], and Andriyanov et al. similarly identified 29 types of ARGs [42]. Tóth et al. tested kefir and yogurt and identified 22 antibiotic resistance genes (ARGs) in the kefir samples, while only 2 ARGs were found in yogurt [43]. In our investigations, a total of 19 ARGs responsible for β-lactamase production were detected in the samples, including two genes (*OXA-662*, *OXA-309*) classified as ESBL genes. Tóth et al. identified a plasmid-borne and MGE *blaZ* gene [17], which Aragao et al. detected in raw goat cheese [44], although we were unable to detect this gene family, we identified a total of five β-lactamase-producing gene families (*ACT*, *TER*, *OXA, ORN*, *CMY*), from which plasmid-borne genes were detected. Of particular concern is the plasmid carriage of the *OXA-662* gene responsible for ESBL production, due to its public health risk. In contrast to our results, Andriyanov et al. identified four different β-lactamase-producing genes (*B-18*, *CME-12*, *GOB-41*, *csp-1*) [42]. Elafify et al. identified *CTX-M-15*, *CTX-M-14*, *SHV-14* genes responsible for ESBL production in raw milk and dairy products [45].

Oniciuc et al. identified *vanRM*, *vanUG*, *vanXYC*, *vanYB* and *vanTC* genes responsible for glycopeptide resistance in raw milk and dairy products [46], but these genes did not reach the identity threshold in our samples. The *tetM* gene responsible for target protection against tetracycline agents was detected in all samples, as Ning et al. also found its presence in raw milk [47]. This gene was plasmid-borne in matured cheese samples and was also found to be an MGE. However, we only detected the efflux pump determinant *tetB* gene in raw milk, whereas Oniciuc et al. additionally identified *tetA* and *tetO* genes [46], and Rodrigues et al. identified the *tetK* gene from raw milk and cheese [48]. Tóth et al. detected the plasmid-borne *tet38* gene responsible for pumping tetracyclines out of cells from raw milk samples, which was also described in another study [17], whereas we identified the *tet33* gene in multiple samples, which was also plasmid-borne. Among the genes responsible for enzymatic inactivation of aminoglycosides, the *AAC*(*6*′)*-IIf* gene was detected in raw milk and matured cheese, while the *AAC*(*6*′)*Ii* and *AAC*(*6*′)*-Iih* genes were only found in matured cheese. The *aadA27*, *APH*(*3*″)*-Ib* and *APH*(*6*)*-Id* genes were primarily found in raw milk, while the *ANT*(*3*″)*-IIc* gene was exclusively observed in raw milk. Ashraf et al. detected the presence of the *AAC*(*6*′)*-APH*(*2*″) gene complex in raw milk and dairy product samples [49], which was also detected by Liu et al. in various animal-origin raw milk samples [50]. Endre et al. demonstrated the presence of the *AAC*(*6*′) gene in raw sheep milk and cheese samples [51]. Parry-Hanson Kunadu et al. detected the *AAC*(*6*′)*-Iy* gene in raw cheese [52].

Among the genes responsible for resistance to fluoroquinolone agents, we identified the efflux pump genes *emrA*, *emrB*, *emrR*, *mdtH*, *patA* and *patB* in all samples. Additionally, we detected the *marA* gene responsible for decreased permeability and efflux pumping in all samples, while the *ramA* gene was only found in raw milk and matured cheese samples. The widely distributed multidrug-resistance efflux pump system and target-modification genes *acR-tolC* and *soxR* were detectable in all samples, similarly to the permeability-reducing *soxS* gene. The *adeF* gene, responsible for developing resistance to fluoroquinolones and tetracyclines through efflux pumps, was detected in all samples, which was also identified by Andriyanov et al. in raw milk samples [42]. The *abaQ* gene, determining resistance to fluoroquinolones via efflux pumps, was only identified in raw milk samples, similar to the findings of Tóth et al. [17].

The horizontal transfer of such resistance genes into the constituents of the human gut microbiome can pose serious public health risks. According to a 2019 report from the Centers for Disease Control and Prevention (CDC), there are 2.8 million infections annually in the USA that can be definitively linked to antibiotic-resistant infections, of which 35,000 cases result in fatal outcomes [53]. Both the gastrointestinal systems of animals and humans can harbor bacteria that may serve as reservoirs of antibiotic resistance, which may originate from intrinsic or extrinsic carriage of ARGs [54]. Intrinsic antibiotic resistance is a natural phenomenon found in all bacterial species, mediated by the bacterial outer membrane and efflux pumps. Resistance can be induced by spontaneous mutations, recombination and horizontal gene transfer, as well as by the use of antibiotics at low doses [55].

Our results indicate that ARGs may not only be present in raw milk but also in raw milk products, and prolonged aging of these products appears to lead to an increase in the frequency of resistance gene sets. This raises the need for further investigations to explain whether the initial gene set originates from the animal organism or is introduced after milking, as well as to critically explore the source of genes observed exclusively in matured cheese samples. The carriage of genes resistant to disinfectants also raises questions regarding the compounds used to disinfect the teats or milking equipment.

Regarding the taxonomic composition, in raw milk, the Firmicutes (Bacillota) phylum was present in 41%, while the Proteobacteria phylum accounted for 58%. In fresh cheese, this ratio changed to 93% and 7%, respectively, and in matured cheese, it was present in 79% and 21%, respectively. Delbes et al. found that the majority of the bacteria in raw milk were Firmicutes (57.6%) and *Clostridiales* (24%) phyla. and that the majority of Firmicutes belonged to the *Lactobacillales* (16%) and *Bacillales* (7.2%) orders [56]. Salazar et al. observed that in commercially available semi-hard cheeses made from pasteurized and raw cows’ milk, the proportions of *Lactococcus* (about 49%, about 40%) and *Bacillaceae* genera (about 48%, about 45%) were similarly low, while the proportion of *Lactobacillus* (about 10%) was dominant in pasteurized milk but not in raw milk, where a higher proportion of *Staphylococcus* (about 4%) was observed [57]. In our study, the proportion of *Lactobacillus* was 3% in raw milk, which increased to 51% in fresh cheese, but decreased to 20% in matured cheese. The proportion of *Bacillaceae* was 0.8% in raw milk, 0.3% in fresh cheese and 0.04% in matured cheese. In contrast to the Salazar et al. study, the proportion of *Staphylococcus* in raw milk was only 0.6%. In a comprehensive metagenomic study of artisanal semi-hard cheeses, the proportion of *Lactococcus* genus was determined to be 84.5% [58]. The differences may be attributed to different cheese cultures used in cheese making or variations in milk microbiomes. For example, Lusk et al. found that in Latin-style cheeses, this proportion ranged from 2–22%, while the *Bacillaceae* family occured in less than 10% [59]. In certain types of cheese, such as Swiss cheese, *Staphylococcus* species are used as starter cultures [60] with a proportion of 0.17% in semi-hard cheeses [58], and less than 3% in Latin cheeses [59].

In our study, *Klebsiella* genus was present in raw milk with a frequency of 4%, and also of 4% in fresh cheese and of 2% in matured cheese. *Klebsiella* genus is a pathogen causing human illness and can also cause spoilage of semi-hard and hard cheeses through gas production; however, it is not heat-resistant, so it can only occur in pasteurized products through contamination [61]. The presence of *Clostridium* genus in products indicates contamination [62]. In an Iranian survey, the microbe was identified in 1.52% of cheese samples [63]. From a public health perspective, identifying the source of contamination is essential, with multiple samples collected at critical points along the production chain. In light of our findings, efforts to reduce antibiotic use closely linked to the development of resistance need to be emphasized even more, striving for alternatives to antibiotics [64] such as antimicrobial peptides [65], propolis [66,67,68,69] or plant essential oils [70]. The widespread presence of genes conferring resistance to cephalosporins—particularly ESBL genes—and fluoroquinolones in the samples is particularly concerning.

Overall, it can be concluded that the majority of bacterial genera identified in this study were present in all three samples; however, differences were observed in the proportions of populations. The lack of microbial suppression in raw milk products may be one possible explanation for the enrichment of resistance genes, which also increases the risk of horizontal gene transfer, and the presence of numerous mobile genetic elements (MGEs) poses an even greater risk. There are natural limiting factors in the transfer of ARGs [71], among which the most important is the shared physical space, known as the minimal critical density of bacterial populations [72]. Another limiting factor is the energy cost associated with carrying individual ARGs and the mechanisms they express, and the maintenance of these genes will only be successful if it confers an evolutionary advantage to the bacteria [71,73,74,75]. Acquisition of resistance genes from the gut microbiome via milk or dairy products can only occur through transformation and transduction if they are pasteurized; however, the high bacterial load in raw milk and dairy products increases the likelihood of conjugation, which poses a much greater risk in the spread of antimicrobial resistance [76]. In addition to the public health significance, the economic and environmental implications should be highlighted, as cattle account for 80% of global dairy production. The emergence of bacteria carrying ARGs can be linked to the cross-treatment of animals with subclinical mastitis through udder fusion. Although this risk can be mitigated by adhering to the sanitary withdrawal period, many farmers do not strictly follow these guidelines. Unable to afford disposing of the milk, they often feed it to calves or simply discard it [77].

Our results are important not only for the general public but also for transferring reliable scientific knowledge through social media platforms [78]. Raising awareness is crucial for educating future veterinarians [79], professionals and farmers, and for building educational programs on this topic. Developing informed consumers, public habits and educational methods is essential for advancing the One Health approach.

## 5. Conclusions

Enhancing health security requires collaborative thinking and research in both animal and public health sectors. Our results highlight the potential risks associated with untreated products and the possible role of ARGs in mediating conjugation between animal products and human pathogens. Our study supports the need for broader investigations with larger sample sizes and sampling at multiple critical points along the food chain. The incorporation of next-generation sequencing into product surveillance offers new perspectives for addressing food safety concerns for future generations. Our findings corroborate the hypothesis that the antibiotic-resistance gene (ARG) content in foods produced through lactic acid fermentation, coagulation and maturation processes may increase due to bacterial proliferation. The data indicate that monitoring and selective use of starting culture strains in such foods are crucial to minimize the presence of ARGs in food products.

## Data Availability

The datasets used and/or analysed during the current study are available from the corresponding author on reasonable request. The sequencing files are available at the LINK below. https://www.ncbi.nlm.nih.gov/bioproject/PRJNA1105066 (accessed on 26 April 2024).

## Supplementary Materials

The following supporting information can be downloaded at: https://www.mdpi.com/article/10.3390/vetsci11060265/s1, Figure S1: Principal component analysis based on read coverage of antimicrobial resistance genes (ARGs) with at least 90% identity and coverage in each sample; Figure S2: Heatmap representation of the read coverage of ARGs with at least 90% identity and coverage in a given sample; Figure S3: Principal component analysis based on read coverage of ARGs with at least 90% identity and coverage in a given sample, only for significant (*p* < 0.05) genes; Figure S4: Plot on heatmap the read coverage of ARGs with at least 90% identity and coverage in a given sample, only for significant (*p* < 0.05) genes; Figure S5: Shannon diversity distribution at genus level between samples using Mann-Whitney test. The comparison shows a significant difference between all groups; Figure S6: The distribution of observed genes, using the Mann-Whitney test, which gives information on the number of genes in a given sample; Figure S7: Non-metric multidimensional scaling (NMDS) ordination of Bray-Curtis distances between samples; Figure S8: Distribution of Shannon diversity (species level) by group. All samples showed significant differences (*p* < 0.05); Figure S9: Distribution of the number of species observed. Comparisons between groups were made using the Mann-Whitney test; Figure S10: Non-metric multidimensional scaling (NMDS) ordination of Bray-Curtis distances between samples; Table S1: The antimicrobial resistance genes (ARGs) identified in the samples, their coverage and identity, and the mechanisms of resistance of each gene to antibiotic groups (continues); Table S2: The antimicrobial resistance genes (ARGs) identified in the samples, their coverage and identity, and the mechanisms of resistance of each gene to antibiotic groups (continues); Table S3: The antimicrobial resistance genes (ARGs) identified in the samples, their coverage and identity, and the mechanisms of resistance of each gene to antibiotic groups (continues); Table S4: The antimicrobial resistance genes (ARGs) identified in the samples, their coverage and identity, and the mechanisms of resistance of each gene to antibiotic groups (continues); Table S5: The antimicrobial resistance genes (ARGs) identified in the samples, their coverage and identity, and the mechanisms of resistance of each gene to antibiotic groups (continues); Table S6: ARG mobility between groups tested by logistic regression between groups.
