# Supplementary material for: Monitoring Changes in the Antimicrobial-Resistance Gene Set (ARG) of Raw Milk and Dairy Products in a Cattle Farm, from Production to Consumption
Source: Vet Sci. 2024 Jun 8;11(6):265. doi: 10.3390/vetsci11060265 (PMC11209563; doi:10.3390/vetsci11060265)
Supplement: Supplementary file 1 [file vetsci-11-00265-s001.zip › vetsci-3009693-supplementary/Supplementary materials.pdf]

Supplementary Table S1. The antimicrobial resistance genes (ARGs) identified in the samples, their coverage and identity, and the mechanisms of resistance of each gene to antibiotic groups

Gene	Coverage	Identity	Group	Mechanism
*AAC(6')-If**	100%	97,22%	aminoglycosides	enzymatic inactivation
*AAC(6')-Ii**	93,41%	98,82%		
AAC(6')-Iih	100%	100%		
*aadA27**	100%	98,85%		
abaQ	101,38%	72,71%	fluoroquinolones	efflux pump
acrA,****	100%	90,68%	fluoroquinolones; cephalosporins; glycylcyclines; penicillins; tetracyclines; rifamycins; phenicols; disinfectants	
acrAB-TolC and *marR mutation**	103,47%	82,64%		target modification, efflux pump
acrB, ****	100%	92,95%		efflux pump
*acrD**	100%	95,08%	aminoglycosides	
acrE	100%	100%	fluoroquinolones; cephalosporins; cephamycin; penicillins	
acrF	100%	99,52%		
acrS	100%	100%	fluoroquinolones; cephalosporins; glycylcyclines; cephamycin; penicillins; tetracyclines; rifamycins; phenicols; disinfectants	
ACT-5	108,92%	99,74%	carbapenems; cephalosporins; cephamycin; penicillins	enzymatic inactivation
*ACT-8**	100%	76,58%		
*adeF**	99,15%	61,15%	fluoroquinolones; tetracyclines	efflux pump
ANT(3'')-IIa,****	83,28%	89,59%	aminoglycosides	enzymatic inactivation
APH(6)-Id,****	100%	99,64%		
*arnT**	100%	86,39%	peptide antibiotics	target mutation
bacA, **, ****	100%	93,41%	peptide antibiotics	target mutation
baeR, ***	100%	94,17%	aminoglycosides; aminocoumarins	efflux pump
baeS	100%	100%		
cmx, ****	100%	100%	phenicols	
CMY-101	100%	99,74%	cephamycin	enzymatic inactivation

* on plasmid; ** on phage; *** mobile genetic element (MGE)

Supplementary Tabel S2. The antimicrobial resistance genes (ARGs) identified in the samples, their coverage and identity, and the mechanisms of resistance of each gene to antibiotic groups (continues)

Gene	Coverage	Identitiy	Group	Mechanism
*CMY-136**	86,88%	94,86%	cephalosporins; cephamycin	enzymatic inactivation
*CMY-59**	107,34%	97,22%	cephamycin	enzymatic inactivation
*CMY-65**	100%	100%	cephamycin	enzymatic inactivation
*CMY-70****	100%	99,48%	cephamycin	enzymatic inactivation
CMY-82	100%	99,48%	cephamycin	enzymatic inactivation
*CMY-83**	98,95%	100%	cephamycin	enzymatic inactivation
*cpxA***	100%	98,03%	aminoglycosides; aminocoumarins	efflux pump
*CRP**	100%	99,05%	macrolides; fluoroquinolones; penicillins	efflux pump
*dfrE**	100%	98,17%	diaminopirimidinek	target replacement
eatAv	100%	99%	pleuromutilinek	target protection
EC-14	102,92%	98,14%	cephalosporins	enzymatic inactivation
efmA	100%	99,77%	macrolides; fluoroquinolones	efflux pump
*efrA**	100%	99,65%	macrolides; fluoroquinolones; rifamycins	efflux pump
EF-Tu	96,33%	94,92%	elfamicin	target mutation
emeA	97,71%	97,71%	disinfectants	efflux pump
*emrB**	100%	95,22%	fluoroquinolones	efflux pump
emrE	100%	98,18%	macrolides	efflux pump
*emrK**	110,26%	99,72%	tetracyclines	efflux pump
*emrR**	100%	94,86%	fluoroquinolones	efflux pump
eptA	101,83%	100%	peptide antibiotics	target mutation
eptB	98,08%	89,89%	peptide antibiotics	target mutation
*evgA**	100%	100%	macrolides; fluoroquinolones; penicillins; tetracyclines	efflux pump
evgS	100%	100%	macrolides; fluoroquinolones; penicillins; tetracyclines	efflux pump
*fosA2**	100%	95,74%	fosfomicin	enzymatic inactivation
*fosA5**	100%	89,93%	fluoroquinolones; aminoglycosides; fosfomycin	enzymatic inactivation
*fosA8**	97,87%	61,76%	fosfomycin	enzymatic inactivation
*gadX**	100%	100%	macrolides; fluoroquinolones; penicillins	efflux pump
glpT	100%	97,35%	fosfomycin	target mutation

* on plasmid; ** on phage; *** mobile genetic element (MGE)

Supplementary Tabel S3. The antimicrobial resistance genes (ARGs) identified in the samples, their coverage and identity, and the mechanisms of resistance of each gene to antibiotic groups (continues)

Gene	Coverage	Identitiy	Group	Mechanism
H-NS	100%	100%	macrolides; fluoroquinolones; cephalosporins; cephamycin; penicillins; tetracyclines	efflux pump
*ICR-Mo**	101,79%	95,6%	peptide antibiotics	target mutation
kdpE	100%	90,18%	aminoglycosides	efflux pump
*kpnE**,***	100%	80,83%	macrolides; aminoglycosides; cephalosporins; tetracyclines; peptide antibiotics; rifamycins; disinfectants	efflux pump
*kpnF**,***	100%	85,32%	macrolides; aminoglycosides; cephalosporins; tetracyclines; peptide antibiotics; rifamycins; disinfectants	efflux pump
kpnG	100%	94,36%		
kpnH	99,8%	84,17%		
*leuO**	102,23%	79,61%	nucleoside antibiotics; disinfectants	efflux pump
lmrD	100%	100%	lincosamides	efflux pump
lptD	100,9%	93,09%	peptide antibiotics; aminocoumarins; rifamycins	efflux pump
lsaA	100%	98,59%	lincosamides; sztreptogramin; streptogramin A; streptogramin B; pleuromutilins	target protection
lsaD	100%	94,57%	lincosamides; streptogramin; streptogramin A; pleuromutilins	target protection
*marA**	100%	93,7%	fluoroquinolones; monobactam; carbapenems; cephalosporins; glycylcyclines; cephamycin; penicillins; tetracyclines; rifamycins; phenicols; penem; disinfectants	efflux pump, permeability reduction
*mdfA**,**	99,76%	86,98%	tetracyclines; disinfectants	efflux pump
mdtA	109,64%	99,52%	aminocoumarins	
mdtB	95,29%	93,24%		
*mdtC**	100%	91,22%		

* on plasmid; ** on phage; *** mobile genetic element (MGE)

Supplementary Tabel S4. The antimicrobial resistance genes (ARGs) identified in the samples, their coverage and identity, and the mechanisms of resistance of each gene to antibiotic groups (continues)

Gene	Coverage	Identitiy	Group	Mechanism
*mdtE**	100%	99,74%	macrolides; fluoroquinolones; penicillins	efflux pump
mdtF	100%	100%		
mdtG	99,51%	90,66%	fosfomycin	
mdtH	100%	100%	fluoroquinolones	
*mdtM**	100,73%	86,73%	fluoroquinolones; lincosamides; nucleoside antibiotics; phenicols; disinfectants	
mdtN	100%	88,92%	nucleoside antibiotics; disinfectants	
mdtO	100%	99,56%		
mdtP	100%	99,8%		
mreA	100,64%	72,61%	macrolides	
*msbA***	100%	92,44%	nitroimidazoles	
*norC**	77,06%	99,16%	fluoroquinolones; disinfectants	
ompA	95,19%	96,07%	peptide antibiotics	permeability reduction
oqxA	100%	91,3%	fluoroquinolones; glycylcyclines; tetracyclines; diaminopirimidinek; nitrofurans	efflux pump
oqxB	100%	96,76%		
*ORN-1**	100%	100%	carbapenems; cephalosporins	enzymatic inactivation
*OXA-309**	97,08%	96,24%	carbapenems; cephalosporins; penicillins	
*OXA-662**	100%	99,27%		
OXY-1-2	100%	100%	monobactams; cephalosporins; penicillins	
OXY-1-4	100%	99,31%		
OXY-6-2	93,1%	100%		
patA	100,89%	67,73%	fluoroquinolones	efflux pump
patB	101,02%	68,24%		
PLA-1	100%	99,66%	carbapenems; cephalosporins	enzymatic inactivation

* on plasmid; ** on phage; *** mobile genetic element (MGE)

Supplementary Table S5. The antimicrobial resistance genes (ARGs) identified in the samples, their coverage and identity, and the mechanisms of resistance of each gene to antibiotic groups (continues)

Gene	Coverage	Identitiy	Group	Mechanism
pmrF	101,55%	84,21%	peptide antibiotics	target mutation
*qacEdelta1** ***	100%	100%	disinfectants	efflux pump
RAHN-1	100%	100%	cephalosporins	enzymatic inactivation
*ramA**,**	91,13%	95,58%	fluoroquinolones; monobactam; carbapenems; cephalosporins; glycylcyclines; cephamycin; penicillins; tetracyclines; rifamycins; phenicols; penem; disinfectants	efflux pump, permeability reduction
rpsL	100%	87,8%	aminoglycosides	target mutation
*rsmA**	100%	85,25%	fluoroquinolones; diaminopyrimidines; phenicols	efflux pump
sepA	71,34%	97,32%	disinfectants	
soxR	100%	100%	fluoroquinolones; cephalosporins; glycylcyclines; penicillins; tetracyclines; rifamycins; phenicols; disinfectants	target modification, efflux pump
*soxS**,**,***	101,87%	89,72%	fluoroquinolones; monobactams; carbapenems; cephalosporins; glicyclines; cephamycin; penicillins; tetracyclines; rifamycins; phenicols; penem; disinfectants	target modification, efflux pump, permeability reduction
*sul1**,***	100%	100%	sulfonamides	target replacement
*TER-2**	101,06%	98,94%	carbapenems; cephalosporins	enzymatic inactivation
*tet33**	95,82%	99,74%	tetracyclines	efflux pump
*tetB**	100%	99,5%		
*tetM**,***	100%	96,4%		target protection
*tetR**	99,52%	100%		target modification, efflux pump
tolC	99,6%	99,8%	macrolides; fluoroquinolones; aminoglycosides; carbapenems; cephalosporins; glycylcyclines; cephamycin; penicillins; tetracyclines; peptide antibiotics; aminocoumarins; rifamycins; phenicols; penem; disinfectants	efflux pump
ugd	100%	98,97%	peptide antibiotics	target mutation
*uhpT****	100%	96,27%	fosfomycin	
yojI	100%	100%	peptide antibiotics	efflux pump

* on plasmid; ** on phage; *** mobile genetic element (MGE)

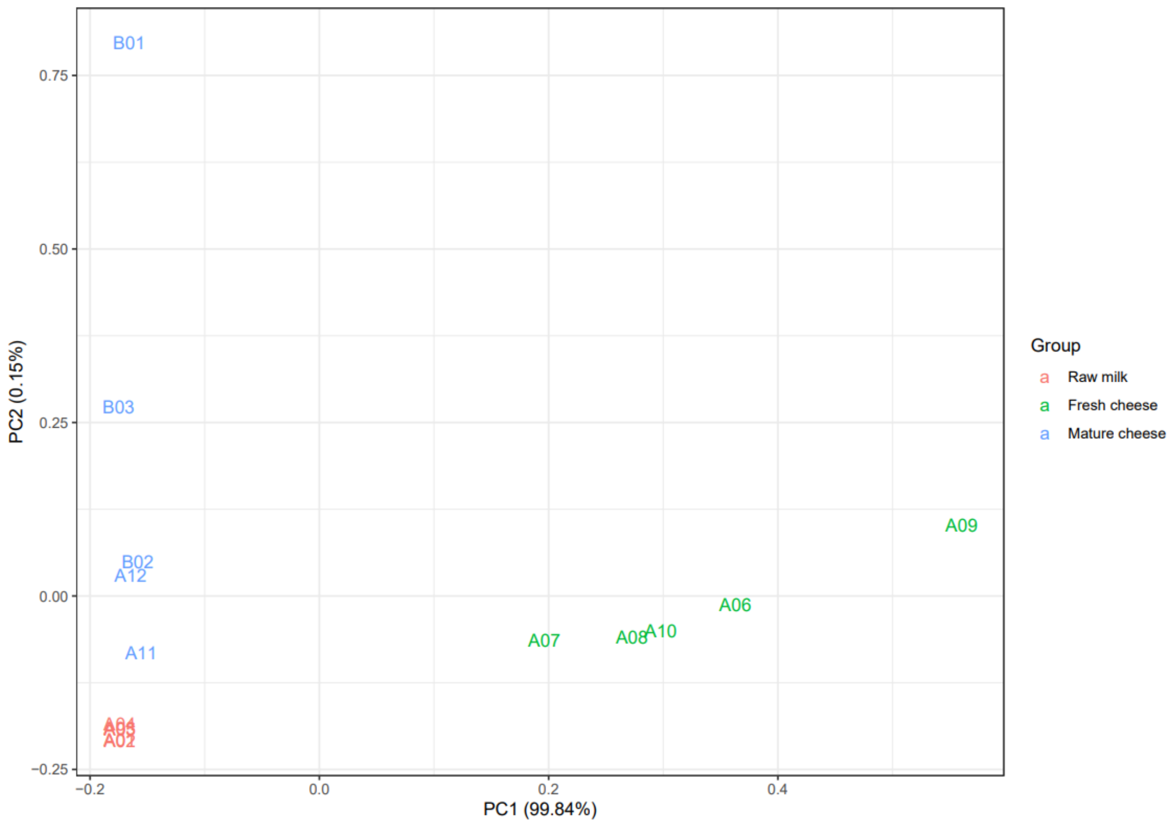

Supplementary Figure S1. Principal component analysis based on read coverage of antimicrobial resistance genes (ARGs) with at least 90% identity and coverage in each sample. The presence of antibiotic resistance genes and their expression in the samples, i.e. the extent to which they cover the entire ARG set, suggests that they contribute slightly to the variance of the samples.

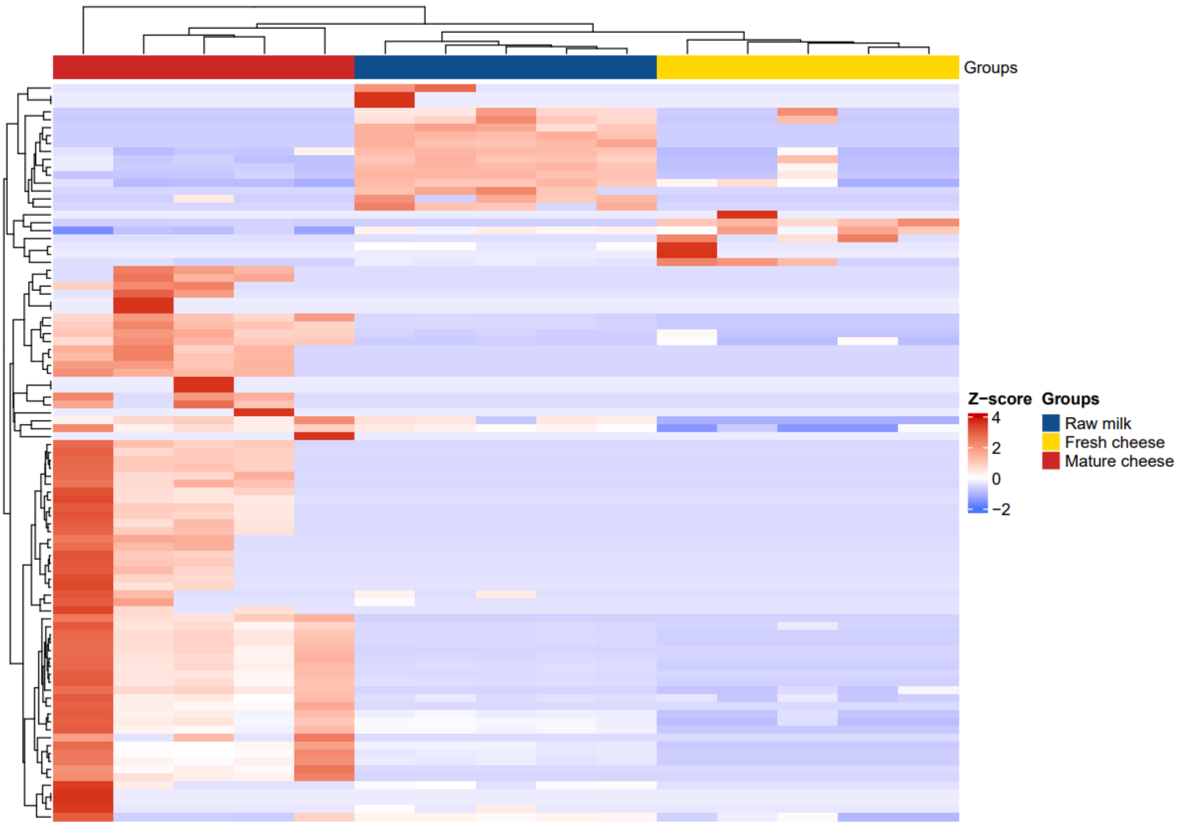

Supplementary Figure S2. Heatmap representation of the read coverage of ARGs with at least 90% identity and coverage in a given sample. Overall, the range of the Z-score from -2 to +4 indicates that the ARG values of a given sample are different and varied, and there may be large differences in the presence or absence of ARGs in the three product types.

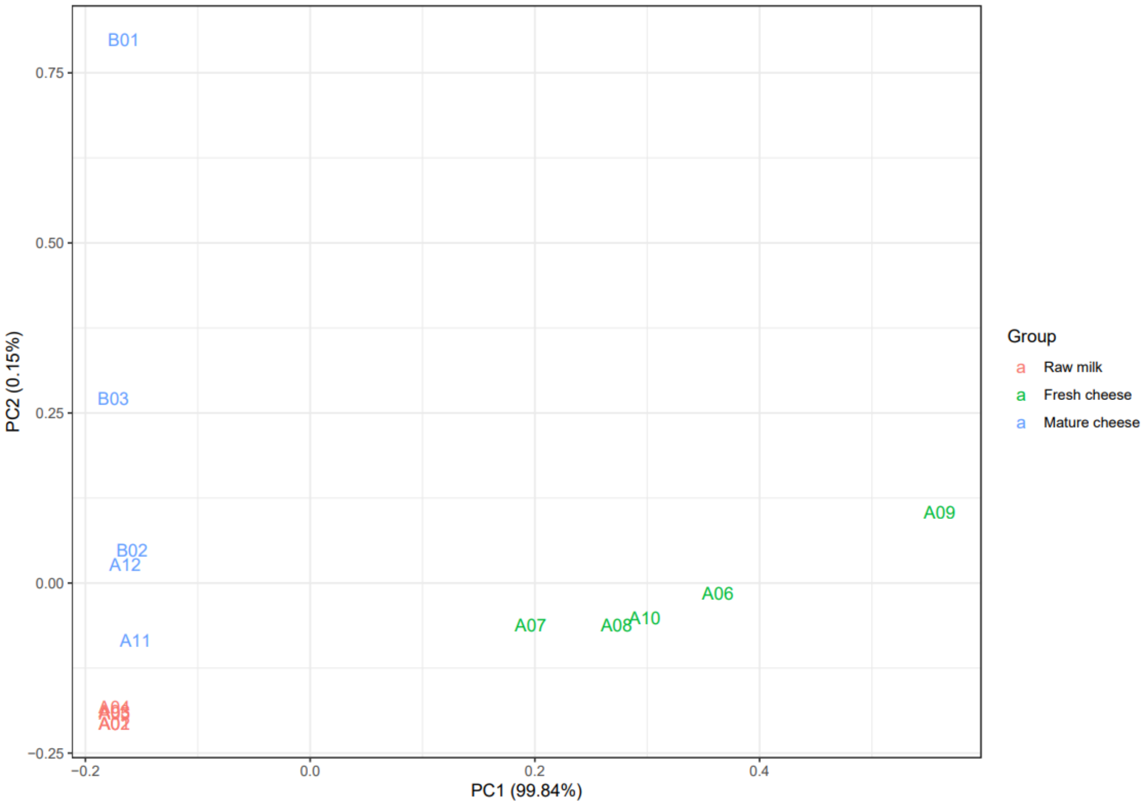

Supplementary Figure S3. Principal component analysis based on read coverage of ARGs with at least 90% identity and coverage in a given sample, only for significant (p<0.05) genes

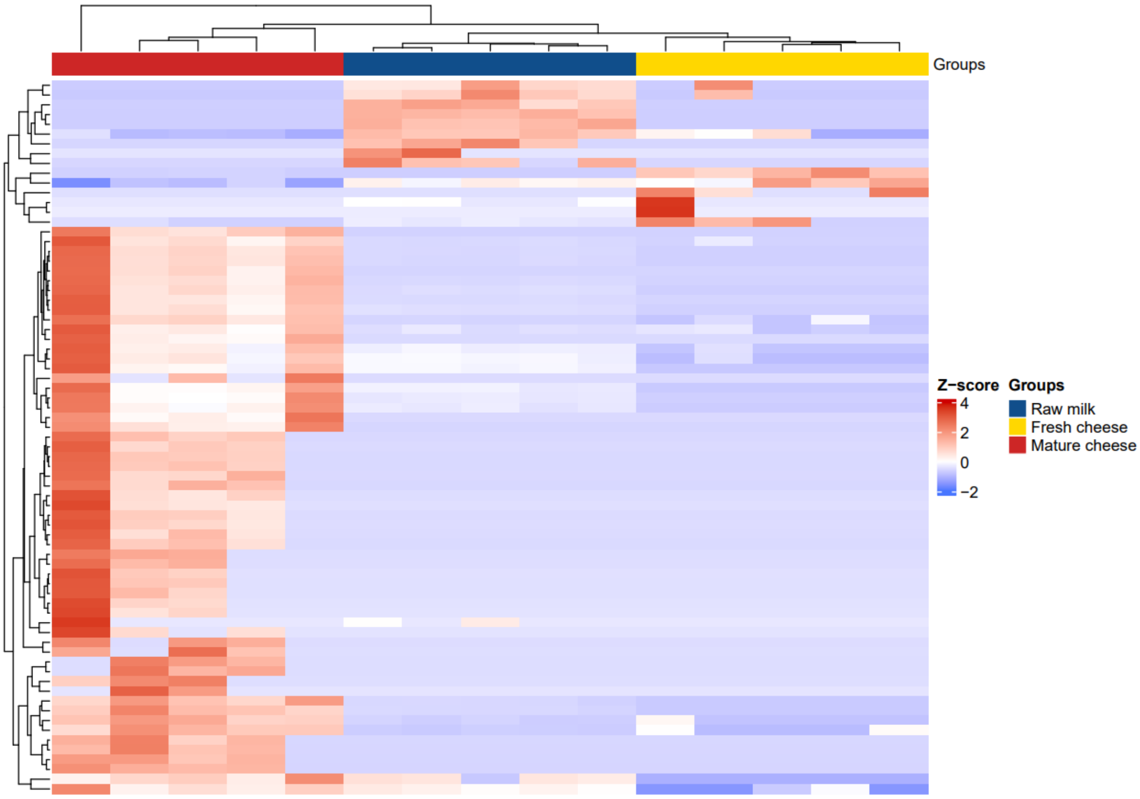

Supplementary Figure S4. Plot on heatmap the read coverage of ARGs with at least 90% identity and coverage in a given sample, only for significant (p<0.05) genes

Supplementary Table S6. ARG mobility between groups tested by logistic regression between groups. The intercept denotes raw milk, which is the reference category. If the Odds Ratio (OR) is greater than 1, it means that the odds of mobility are higher, if less than 1, the odds of mobility are lower in that category.

Group	OR	Standard Error	p-value
Intercept	0.168	0.174	$1.456*10^{-24}$
Fresh cheese	2.413	0.355	0.013
Mature cheese	0.605	0.247	0.042

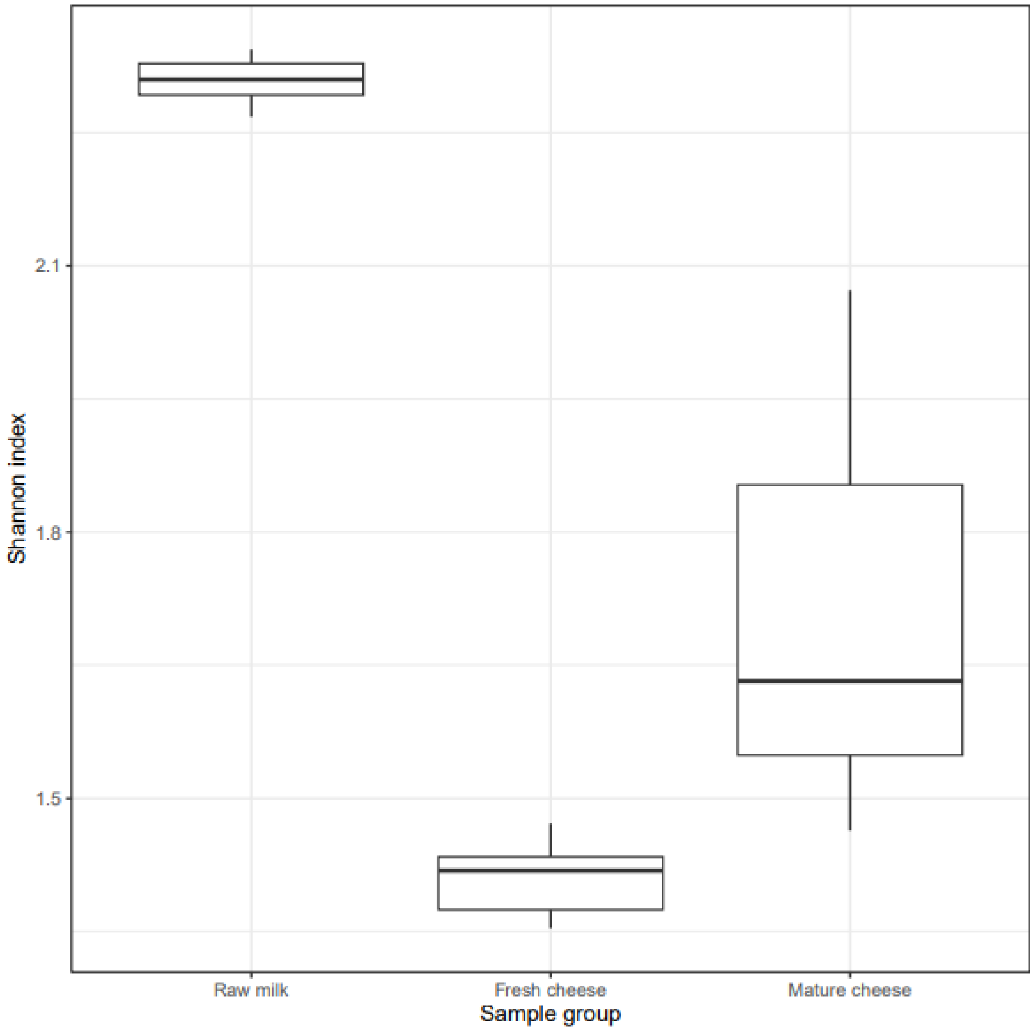

Supplementary Figure S5. Shannon diversity distribution at genus level between samples using Mann-Whitney test. The comparison shows a significant difference between all groups. For raw milk, a high value indicates that many different genus are present in the sample, while for fresh cheese and mature cheese, a lower value indicates that fewer genus are present.

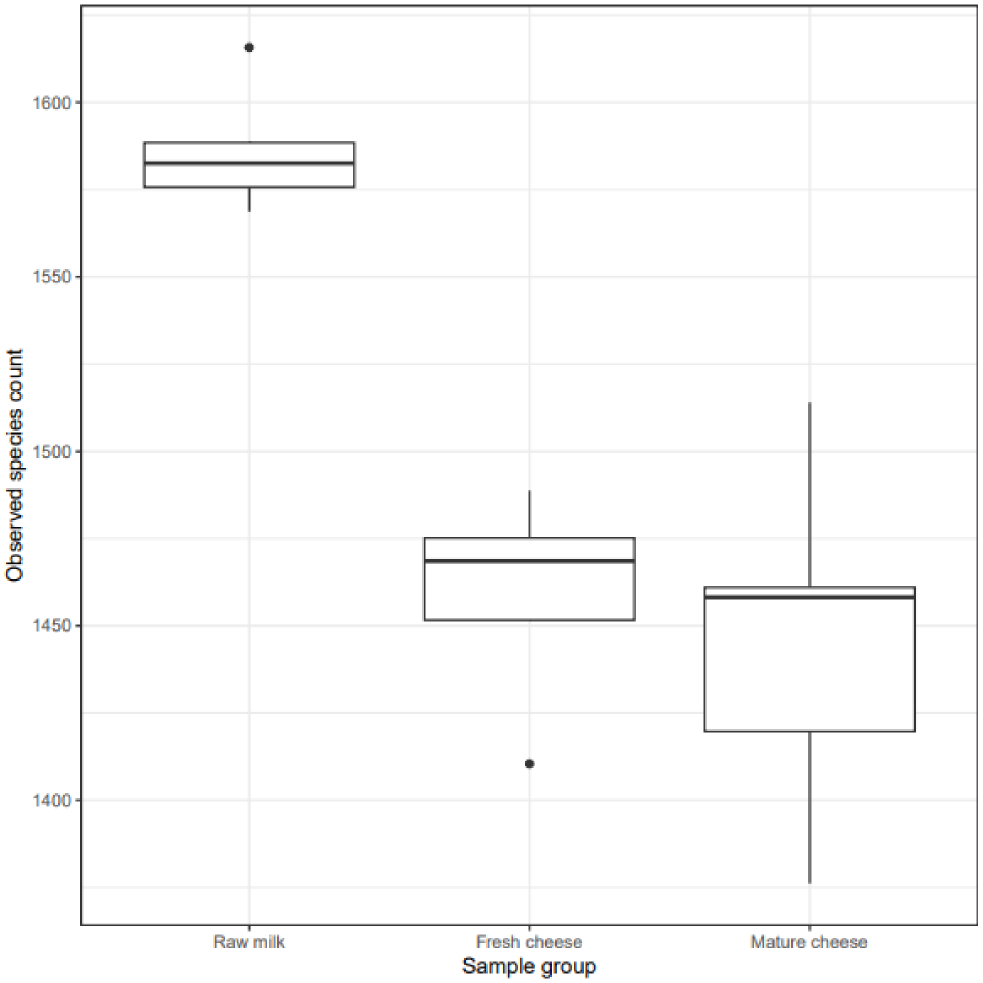

Supplementary Figure S6. The distribution of observed genes, using the Mann-Whitney test, which gives information on the number of genes in a given sample.

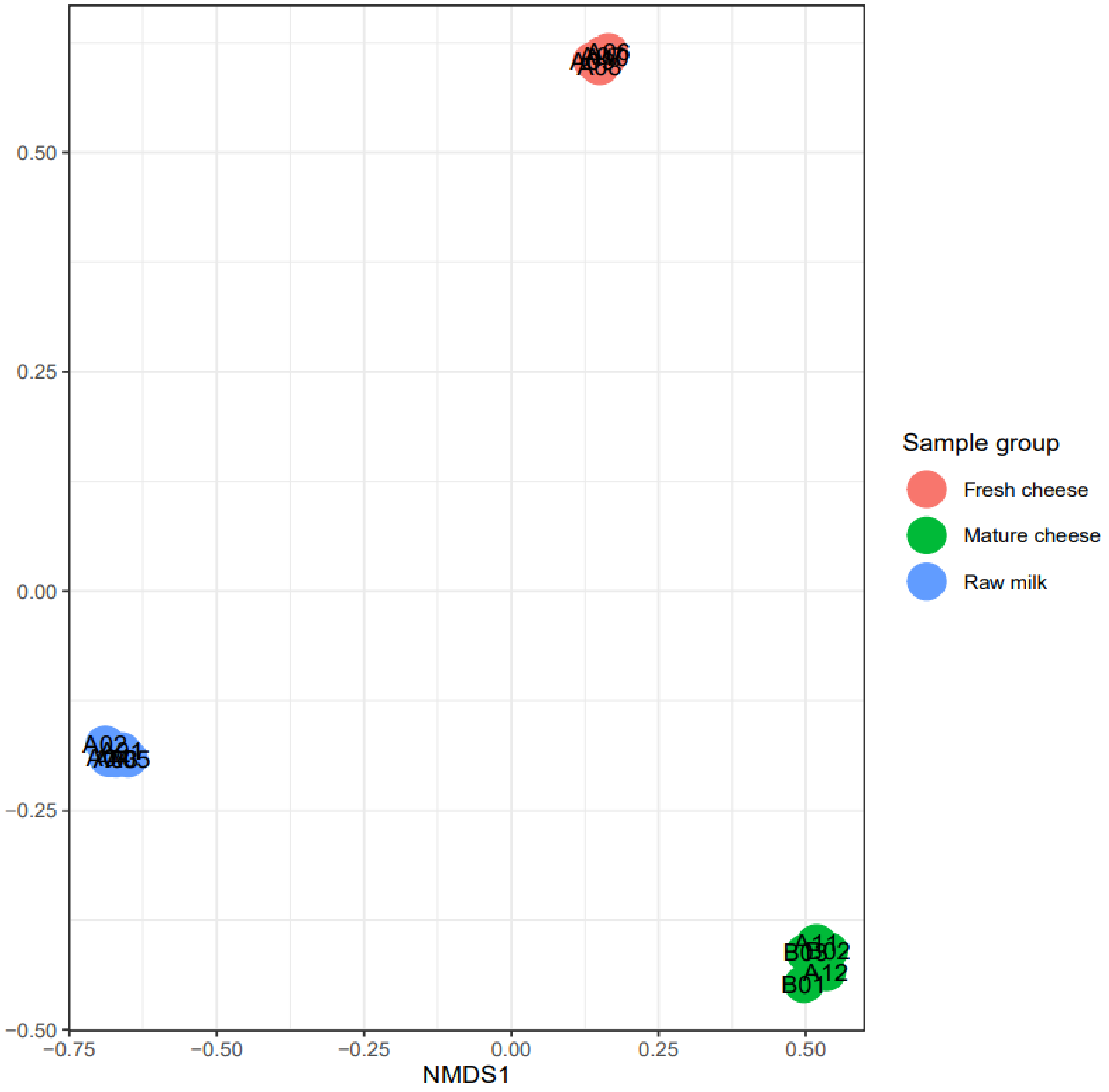

Supplementary Figure S7. Non-metric multidimensional scaling (NMDS) ordination of Bray-Curtis distances between samples. P-value of the permanentova test for differences between samples: 0.001. Differences between samples are not random, they are significantly different.

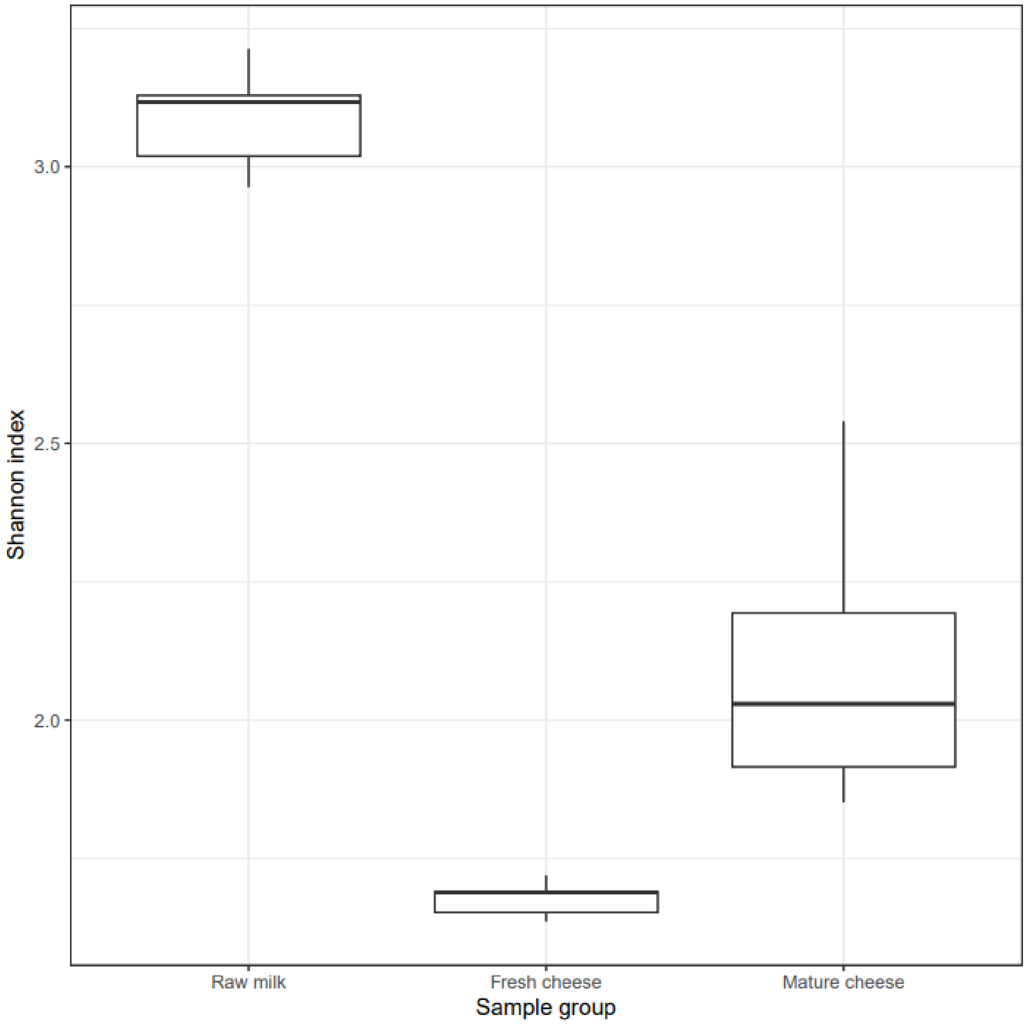

Supplementary Figure S8. Distribution of Shannon diversity (species level) by group. All samples showed significant differences (p<0.05).

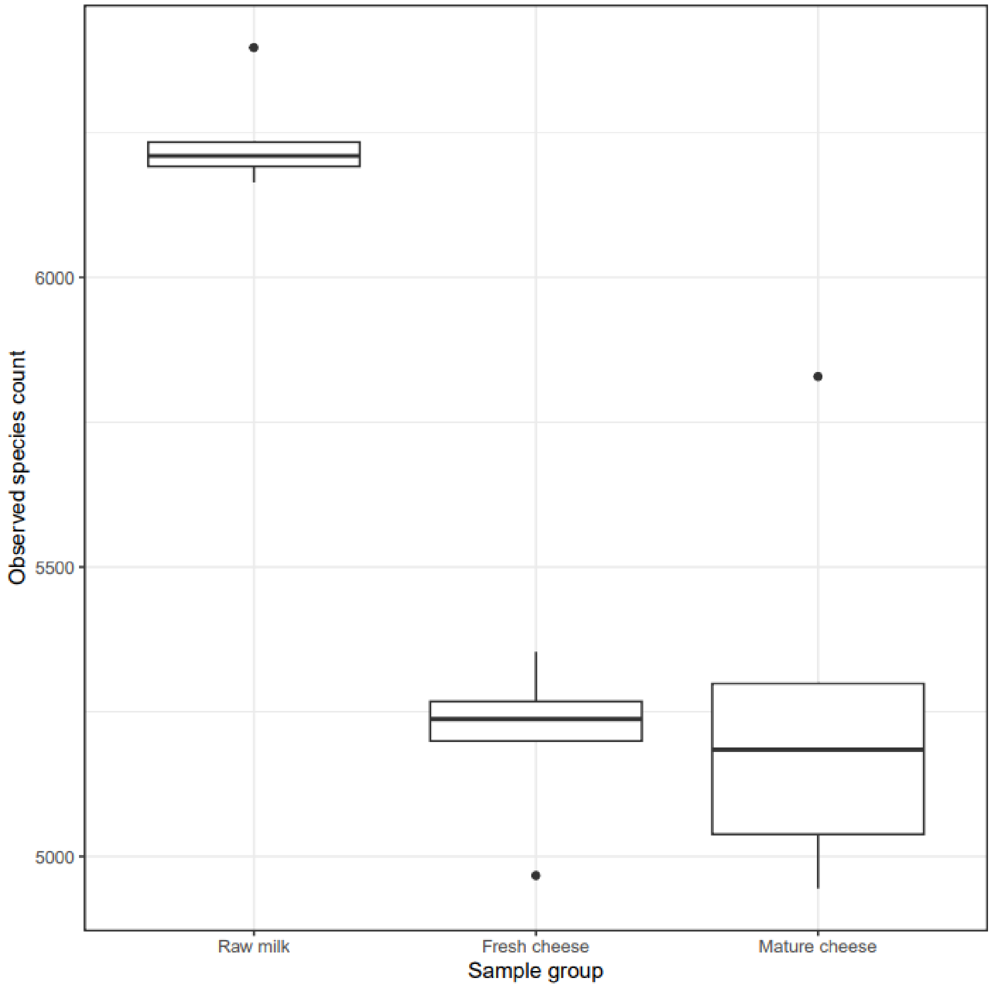

Supplementary Figure S9. Distribution of the number of species observed. Comparisons between groups were made using the Mann-Whitney test.

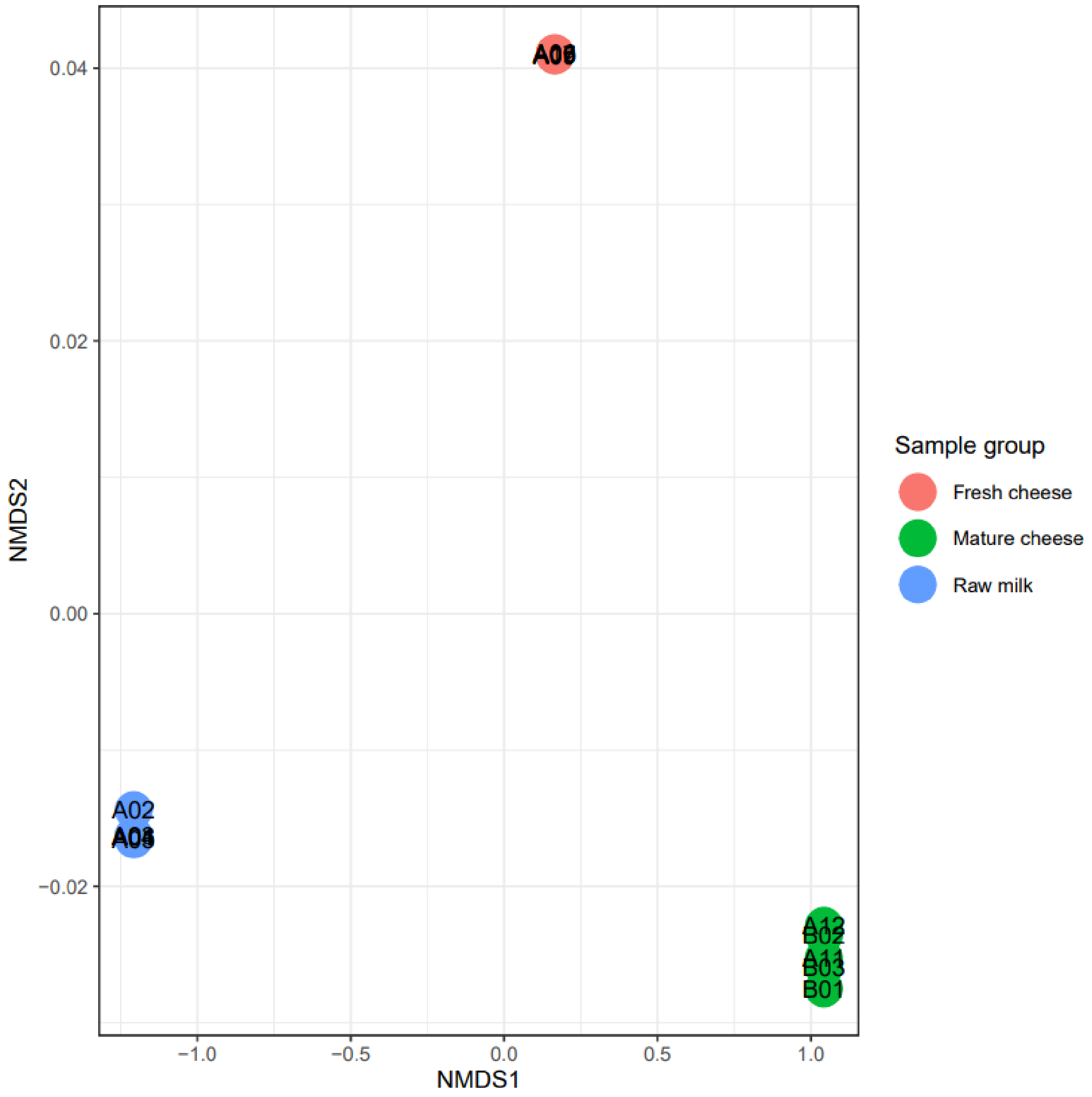

Supplementary Figure S10. Non-metric multidimensional scaling (NMDS) ordination of Bray-Curtis distances between samples. Permanova test for differences between samples p-value: 0.001.
